# Enteric tube placement in patients with esophageal varices: Risks and predictors of postinsertion gastrointestinal bleeding

**DOI:** 10.1002/jgh3.12255

**Published:** 2019-09-10

**Authors:** Lolwa N Al‐Obaid, Ahmad Najdat Bazarbashi, Margot E. Cohen, Judith Kim, Yuxiu Lei, Jordan E. Axelrad, Alyson Fox, Subani Chandra, Fredric D. Gordon

**Affiliations:** ^1^ Department of Internal Medicine Lahey Hospital and Medical Center Burlington Massachusetts USA; ^2^ Department of Medicine New York‐Presbyterian/Columbia University Medical Center New York City New York USA; ^3^ Division of Pulmonary and Critical Care Medicine Lahey Hospital and Medical Center Burlington Massachusetts USA; ^4^ Division of Gastroenterology and Hepatology New York‐Presbyterian/Columbia University Medical Center New York City New York USA; ^5^ Division of Pulmonary and Critical Care Medicine New York‐Presbyterian/Columbia University Medical Center New York City New York USA; ^6^ Division of Transplantation and Hepatobiliary Diseases Lahey Hospital and Medical Center Burlington Massachusetts USA

**Keywords:** cirrhosis, enteric tubes, esophageal varices, gastrointestinal bleeding

## Abstract

**Background and Aim:**

Enteric tube (ET) placement is approached with caution in patients with esophageal varices (EV) due to concern of causing variceal bleeding. Data are limited on rates and predictors of gastrointestinal bleeding (GIB) in these patients. This study aims to assess the rate and predictors of bleeding from EV after ET placement.

**Methods:**

We performed a retrospective chart review on patients requiring ET access with known EV. Inclusion criteria were age >18 with endoscopically proven EV who required ET placement. Patients who were admitted with, or developed a GIB prior to placement of ET were excluded, as were patients admitted for liver transplantation. Primary outcome was incidence of GIB within 48 h of tube placement. Secondary outcome was a >2 g/dL drop in hemoglobin within 48 h of placement without evidence of bleed. Statistical analysis was performed using Fischer's exact test, Mann–Whitney *U* test, and univariate logistic regression model.

**Results:**

A total of 75 patients were included in the analysis. The most common etiology of cirrhosis was alcohol (44%). The most common location of EV was in the lower third of the esophagus (61%). The primary outcome was observed in 11 (14.6%) patients. The secondary outcome was found in eight (10.6%) patients. On univariate analysis, GIB was associated with higher MELD‐Na (*P* = 0.026) and EV located in the lower third of the esophagus (*P* = 0.048).

**Conclusion:**

ET placement in patients with EV is associated with low risk of bleeding. Elevated MELD‐Na and lower EV location conferred a higher risk of bleeding after ET placement.

## Graphical abstract

Enteric tube placement in patients with esophageal varices (EV) is associated with low risk of bleeding. Elevated MELD‐Na and lower EV location conferred a higher risk of bleeding.

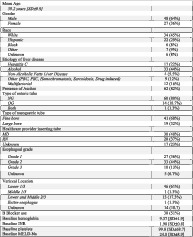

## Introduction

Esophageal varices (EV) are a complication of portal hypertension and are prevalent in about 30% of patients with compensated cirrhosis and 60% of those with decompensated cirrhosis. They carry an annual spontaneous bleeding rate of around 12% and a mortality rate of 15–20%.^1^ It is also estimated that 70% of upper gastrointestinal bleeds in patients with cirrhosis are due to EV bleeds.^2^ Historically, the general practice has been to avoid instrumentation in the esophagus in patients with cirrhosis. Historically, EV have been considered a relative contraindication to gastroscopy.^2^ Esophagogastroduodenoscopy (EGD) now plays an essential role in both the diagnosis and treatment of EV. The role of enteric access in patients with cirrhosis with EV is similarly one of importance; however, its risks require a more thorough evaluation.

Malnutrition in chronic liver disease is seen in 20% of patients with compensated cirrhosis and 60% of those with decompensated cirrhosis.^3^ Malnutrition is characterized as a protein–energy malnutrition (PEM) and is associated with poor clinical outcomes.^4^ PEM has been associated with higher mortality rates, bleeding EV, and refractory ascites, all of which improve with increasing nutrition intake.^3^ In fact, nutrition is so vital to the patient with cirrhosis that the recommendation is for feeding to be resumed as soon as patients have been bleed free for 24 h.^5^ Some patients, however, are unable to meet their nutritional intake requirements for a variety of reasons, such as anorexia or encephalopathy, at which time enteral feeding would be considered. Parenteral nutrition has provided an alternative for nutritional input in cases where enteric access is contraindicated or cannot be established; however, it is associated with various complications, including bloodstream infections.^6^ Another common scenario encountered in clinical practice occurs in patients admitted with hepatic encephalopathy who are at high risk of aspiration and are therefore unable to safely take various medications for the management of complications of portal hypertension, such as lactulose and rifaximin. Despite the availability of IV and rectal formulations of various medications, establishing enteric access in these patients is imperative.

The lack of clarity on the risks of enteric tube (ET) placement in this patient population poses a particular dilemma for health‐care providers. Resident trainees and other health‐care providers in various specialties are often asked to place nasogastric or orogastric tubes in patients with known EV at bedside without knowledge of the risks associated with blind placement. What is known is that placement of enteric feeding tubes in patients with EV is generally approached with caution due to the concern that bedside blind placement of an ET may trigger a variceal bleed by way of blunt trauma to the varix. The data surrounding this topic have been limited, with no data available on risks and predictors of gastrointestinal bleeding (GIB).

## Methods

We performed a retrospective chart review on patients requiring ET access (orogastric or nasogastric) with known EV at two academic liver transplant centers. At one academic medical center, the timeframe for data collection ranged from January 2010 to January 2017. At another academic medical center, the timeframe ranged from 2015 to 2018. Approval for this study was obtained from the institutional review boards of the participating institutions.

### *Inclusion and exclusion criteria*

Inclusion criteria were patients aged >18 years with a history of cirrhosis and endoscopically proven EV who required nasogastric or orogastric tube placement after presenting to the hospital with a non‐GIB chief complaint. Patients who were admitted with or developed a GIB during their inpatient stay prior to placement of an ET were excluded, as were patients with EV who underwent ET placement during admission for liver transplantation.

### *Data collection and outcome measures*

Data were collected on baseline demographics (age, gender), etiology of liver disease, endoscopic grade and location of EV, use of nonselective beta blocker for EV prophylaxis, previous EV bleed or banding, history of encephalopathy, presence of ascites, baseline labs (platelet count, international normalized ratio [INR]), and model of end‐stage liver disease (MELD‐Na) score. The primary outcome was incidence of GIB, defined as hematemesis, bloody NG aspirate, or melena within 48 h of ET placement. The secondary outcome was a >2 g/dL drop in hemoglobin (Hb) within 48 h of ET placement without evidence of bleed. We define a clinically significant drop in hemoglobin as >2 g/dL.

### *Statistical analysis*

Continuous variables are reported as the means ± SD, while categorical variables are reported as the numbers with percentages (%).

Categorical variables were compared using Fisher's exact test and Chi square test as appropriate, while continuous variables were compared using the Mann–Whitney *U* test (Wilcoxon test).

Predetermined *P* values of <0.05 were considered statistically significant. Analysis was performed using the Statistical Package for Social Sciences (SPSS version 24.0.0; IBM SPSS statistics, IBM Corporation, Armonk, NY, USA).

## Results

A total of 75 patients were included in the analysis. Baseline characteristics are summarized in Table 1. Mean age was 59.2 (SD ± 9.9) years; 64% of patients were male. The most common cause of cirrhosis and portal hypertension was alcohol related (44%) followed by hepatitis C (22%). Mean MELD‐Na score was 24.8 (±8.9). Nasogastric tubes (NGT) were the most common type of ETs placed (60%). The most common type of nasogastric tube placed was fine bore (68%, 41/60). The majority of providers placing ETs were physicians (30%, 40/75); however, in 24% of tube placements, the type of provider was not known. One patient required placement of both nasogastric and orogastric tube simultaneously. The most common grade of EV encountered was grade 2 at 44% (33/75), while the most common site for EV development was the lower third of the esophagus at 66% (46/75).

**Table 1:** Baseline characteristics

Mean age	
59.2 years (SD **±** 9.9)	
Gender	
Male	48 (64%)
Female	27 (36%)
Race	
White	34 (45%)
Hispanic	22 (29%)
Black	6 (8%)
Other	7 (9%)
Unknown	6 (8%)
Etiology of liver disease	
Hepatitis C	17 (22%)
Alcohol	33 (44%)
Non‐alcoholic fatty liver disease	4 (5.5%)
Other (PBC, PSC, hemochromatosis, sarcoidosis, drug induced)	9 (12%)
Multifactorial	12 (16%)
Presence of ascites	62 (82%)
Type of enteric tube	
NG	60 (80%)
OG	14 (18.7%)
Both	1 (1.3%)
Type of nasogastric tube	
Fine bore	41 (68%)
Large bore	19 (32%)
Health‐care provider inserting tube	
MD	30 (40%)
RN	28 (37%)
Unknown	17 (23%)
Esophageal grade	
Grade 1	27 (36%)
Grade 2	33 (44%)
Grade 3	10 (13%)
Unknown	5 (6.7%)
Variceal location	
Lower 1/3	46 (61%)
Middle 1/3	1 (1.3%)
Lower and middle 2/3	13 (17.3%)
Entire esophagus	1 (1.3%)
Unknown	14 (18.7)
B blocker use	38 (51%)
Baseline hemoglobin	9.37 (SD ± 1.9)
Baseline INR	1.98 (SD ± 0.8)
Baseline platelets	99.8 (SD ± 68.7)
Baseline MELD‐Na	24.8 (SD ± 8.9)

INR, international normalized ratio; MD, medical doctor; MELD, model for end‐stage liver disease; NG, nasogastric; OG, orogastric; PBC, primary biliary cholangitis; PSC, primary sclerosing cholangitis; RN, registered nurse.

The primary outcome of GIB was seen in 11 (14%) patients. The secondary outcome of a >2 g/dL drop in Hb was seen in nine (12%) patients. Of the variables analyzed, location of EV and MELD‐Na score were both associated with increased risk of bleeding. EV located in the lower esophagus demonstrated an increased risk of bleeding (odds ratio [OR] = 3.16; confidence interval [CI] 0.63–15.9, *P* = 0.0487), more so than varices in any other location of the esophagus. An increasing MELD score was also associated with an increased risk of bleeding, (*P* = 0.0263), with an OR 1.1 (CI 1.01–1.19) for each 1 point increase in MELD‐Na.

## Discussion

In our study of 75 patients with EV who underwent ET placement, the risk of upper GIB was observed to be low at 14%. The predictors of bleeding within the cohorts studied included more advanced liver disease (as documented by higher mean MELD‐Na scores) and location of EV in the lower third of the esophagus. Despite no difference in mean INR and platelet levels between cohorts that experienced GIB and those that did not, we believe that the higher mean MELD‐Na scores contributed to higher risks of bleeding due to various factors, including worse renal function through uremia‐induced platelet dysfunction,^7^ more advanced portal hypertension, and subsequent higher risk of spontaneous bleeding from EV. We hypothesize that the increased risk of bleeding from EV in the lower third of the esophagus stems from the blunt trauma of the ET passing through an inherently narrower gastroesophageal (GE) junction made worse by the thinner nature of the lower EV walls. Our study, despite its sample size limitation, demonstrated no difference in type of ET placed or health‐care provider placing the ET. In addition, we demonstrated that prior B‐blocker use had no impact on the risk of GIB in this population (Table 2).

**Table 2:** Predictors of gastrointestinal bleeding

Predictors	No GI bleed	GI bleed	Odds ratio (CI)	*P* value
EV grade, *n* (%)	0	0	0	0.3303†
Grade 1	30/58 (52)	3/11 (27)	0	0
Grade 2	21/58 (36)	6/11 (55)	0	0
Grade 3	7/58 (12)	2/11 (18)	0	0
EV location, *n* (%)	0	0	0	0.0487†
Lower third	37/63 (59)	9 (82)	0	0
Middle third	1/63 (2%)	0 (0)	0	0
Lower and middle third	11/63 (17)	1 (9)	0	0
Entire esophagus	0/63 (0%)	1 (9)	0	0
Unknown	14/63 (22%)	0 (0%)	0	0
Home B‐blocker, *n* (%)	33/63 (52)	3/11 (27)	0	0.1898‡
Prior banding	14/64 (22%)	3/11 (27%)	0	0
Baseline INR, mean (SD)	2.0 (0.8)	2.1 (0.5)	1.26 (0.6–2.67)	0.1210§
Baseline Plt, mean (SD)	105 (45–165)	97 (26–158)	0	0.7483¶
Baseline MELD‐Na, mean (SD)	24 (9)	30 (7)	1.1 (1.01–1.19)	0.0263^†^
Type of enteric tube, *n* (%)	0	0	0	0.5955^†^
NG	49 (78)	10 (91)	0	0
OG	13 (21)	1 (9)	0	0
Both	1 (1)	0 (0)	0	0
Type of NGT, *n* (%)	0	0	0	0.1446^‡^
Fine bore	31/49 (63)	9/10 (90)	0	0
Large bore	18/49 (37%)	1/10 (10)	0	0
Provider placing tube, *n* (%)	0	0	0	0.4255^†^
MD	24 (38)	6 (55)	0	0
RN	23 (37)	4 (36)	0	0
Unknown	16 (25)	1 (9)	0	0

† Chi‐square test.

‡ Fisher's exact test.

§ Unpaired student's *t* test.

¶ Wilcoxon two‐sample test.

CI, confidence interval; EV, esophageal varices; GI, gastrointestinal; INR, international normalized ratio; MD, medical doctor; MELD, model for end stage liver disease; NG, nasogastric; NGT, nasogastric tube; OG, orogastric; RN, registered nurse.

It is important to note that this study is the first in 30 years to evaluate the risk of upper GIB in patients with EV who had ETs placed. There have been multiple studies that delineate the risks of ET placement in the general population. Depending on the method of placement (unguided *vs* guided), each method carries its own risk profile. Bedside, unguided placement is the most common technique and can be complicated by malpositioning (0.5–16%), with tracheal, pulmonary, or pleural placement in 0.3–15%.^8^ Other complications that may occur, independent of the presence of EV, include tube obstruction, perforation of intestinal tract, intestinal obstruction, mucosal erosions or ulcerations, and hemorrhage among others.^9^

Fine bore NG tubes have reduced the incidence of some complications such as rhinitis, esophageal stricture, and reflux but have not been shown to reduce the other complications mentioned above.^10^ The notion that ETs potentially posed an increased risk of EV bleeds was anecdotal as only one study was observed in the literature. This study, by Ritter *et al*. at the Mayo Clinic from 1985 to 1987, evaluated ET placement in 75 patients with known EV during orthotopic liver transplantation.^11^ Their results demonstrated a <4.8% risk of bleeding from esophageal instrumentation during a surgical procedure.

Despite this being one of very few studies addressing a common clinical question, we acknowledge a few limitations that we hope future studies can address. Our study did not elaborate on a specific time interval between diagnosis of EV and placement of ET; however, we found that the majority of patients did have EGDs performed within 2 years of ET placement. This limitation does not thoroughly address the status of EV that may have transformed with time. Despite the goal of this study to highlight the risk or absence of bleeding, we can not elaborate on the need for endoscopy for individuals who had GIB and their outcomes. However, we do report that only two patients (2.7%) who had experienced a bleed postET placement suffered a Hb drop of more than 2 g/dL, which we defined as a clinically significant Hb drop. We hope that further studies can distinguish between clinically and nonclinically significant GIB postET insertion in patients with EV.

We believe that our study provides insight into a common clinical question asked by health‐care providers caring for patients with cirrhosis. It provides insight into the risks and predictors associated with a commonly performed procedure in a notoriously vulnerable patient population. We hope this study provides a platform for providers to weigh the risks and benefits of ET placements in a subset of at‐risk patients while easing concerns of performing this procedure in lower‐risk patients. We hope this study provides the impetus for further studies to be carried out addressing this question, including larger multicenter studies and prospective studies.
